# Developing a Virtual Reality Simulation Program for Improving Nursing Students’ Clinical Reasoning Skills in Home Settings: A Protocol Paper

**DOI:** 10.3390/nursrep12040093

**Published:** 2022-12-06

**Authors:** Kyoko Yoshioka-Maeda, Chikako Honda, Yuka Sumikawa, Yuko Okamoto, Megumi Shimada, Hitoshi Fujii, Riho Iwasaki-Motegi, Takahiro Miura, Mai Otsuki

**Affiliations:** 1 Department of Community Health Nursing, Division of Health Sciences and Nursing, Graduate School of Medicine, The University of Tokyo, Tokyo 113-0033, Japan; 2 Department of Gerontological Home Care and Long-Term Care Nursing/Palliative Care Nursing, Graduate School of Medicine, The University of Tokyo, Tokyo 113-0033, Japan; 3 Department of Nursing Sciences, Graduate School of Human Health Sciences, Tokyo Metropolitan University, Tokyo 116-8551, Japan; 4 Department of Medical Statistics, School of Nursing, Mejiro University, Saitama 339-8501, Japan; 5 Department of Health Promotion, National Institute of Public Health, Saitama 351-0197, Japan; 6 Human Augmentation Research Center, National Institute of Advanced Industrial Science and Technology, Chiba 277-0882, Japan

**Keywords:** clinical judgment, community, nursing students, randomized controlled trial, simulation, virtual reality

## Abstract

Most nursing simulation programs focus on persons’ healthcare needs in hospital settings, and little is known about how to identify them in home settings. This study aims to develop and validate a virtual reality (VR) simulation program for nursing students to improve their clinical reasoning skills and confidence in assessing persons’ healthcare needs in home settings. We developed a VR simulation program based on a literature review and expert discussion. In Phase 1, home visit nurses or public health nurses will validate the program through their interviews in 2022. In Phase 2, we will conduct a pilot and main single-blinded randomized trial for nursing students to confirm the effectiveness from 2022 and 2023. Participants will be randomly allocated into an intervention group using VR simulations and a control group receiving videos regarding three kinds of community residents’ lives [1:1]. After obtaining informed consent, the students will submit their anonymous data to the researchers to prevent associating their grade evaluation. The primary outcome will be their clinical reasoning skills. The second outcome will include their satisfaction and self-confidence. This study will examine the effectiveness of improving their clinical reasoning skills and confidence in assessing persons’ healthcare needs in home settings.

## 1. Introduction

A simulation program enhances nursing students’ clinical reasoning skills and self-confidence [1]. Simulation training improves nursing students’ knowledge and skills without patients facing any risk [2]. Most simulation training programs for nursing students are designed for the hospital setting and provide training in assessing the patient’s clinical condition and providing appropriate care for them [3]. Additionally, fidelity is essential in improving the effectiveness of simulation training programs [4,5]. Therefore, high-fidelity simulation programs regarding hospital settings enhance nursing students’ competencies and self-confidence. However, previous studies have focused on their clinical reasoning skills in hospital settings [3,6]. Little is known about how to identify persons’ healthcare needs in home settings by nursing students.

Humanoid mannequins with computer-controlled physiological responses and standardized patients are one of the most popular high-fidelity simulation measures, which need massive funds and an extended preparation time [7]. Faculty members seek user-friendly devices, and virtual reality (VR) is a ubiquitous technology for simulation education [8,9]. VR is a computer technology with a sense of immersion and develops an interactive three-dimensional (3D) world [1,10]. Head-mounted display (HMD) is positioned on the head of the user and shows 3D images with immersive experiences [11]. The literature review indicates that VR simulation improves nursing students’ knowledge and self-confidence [1,9,12], and their high-risk newborn infection control educational programs [13].

Additionally, among VR simulations, eye tracking is a novel measure that shows us the users’ attention and traces of gaze [14]. Previous studies show that nursing students’ eye-tracking data is helpful for their clinical decision-making in a hospital setting [15]. However, most simulations focusing on community-based home care settings are not using VR with an eye-tracking system [16,17]. Patients’ health condition is associated with their home settings and lives [6,18]. VR simulation allows them to understand the patient’s home environment and life through 3D images. It is suitable for them to enhance their clinical reasoning skill in home settings for providing needs-oriented care for patients.

In Japan, the national government revised the designated rules of educational institutions for nursing education and stressed the importance of community and home care nursing [19]. The curriculum promotes nursing students’ confidence and desire to start their careers in community settings [20]. They understand the community residents’ lives and environmental association through home visiting practices [21]. Acquiring home visiting skills is essential for bridging the gap between theoretical knowledge and real-world practices [22]. They are expected and required to work as full-fledged professionals immediately [23].

Additionally, the national government has promoted nursing simulation education since 2017 [24]. The coronavirus disease 2019 (COVID-19) pandemic has temporally interrupted nursing education [25] and transformed hands-on traditional nursing education into remote and virtual simulation nursing [26,27]. However, they have enhanced their learning through VR simulation experiences [28], but some need help using it [29]. User-friendly VR simulation would be needed to enhance their clinical reasoning and assess the health needs of community residents in home settings. Therefore, the study aims to develop and validate a VR simulation program for nursing students to improve their clinical reasoning skills and confidence in assessing persons’ healthcare needs in home settings. A pilot and main randomized controlled trial (RCT) will also confirm the effectiveness of the program using relevant scales.

## 2. Materials and Methods

### 2.1. Methodology and Design

We developed a VR simulation program based on the literature review [12,30,31,32] and expert discussion. This study will be conducted in Tokyo, Japan, with two phases. In Phase 1, home visit nurses (HVNs) or public health nurses (PHNs) will validate the VR simulation program, including the goals, simulation scenario, and each clip through interviews in 2022. Based on their comments, we will effectively revise the programs and materials. Additionally, we will record their eye-tracking as data with their permission to compare for the students.

In Phase 2, we will conduct a pilot and main RCT for nursing students to confirm the effectiveness from 2022 to 2023. This study will be a two-armed single-blinded RCT.

**Figure 1:**
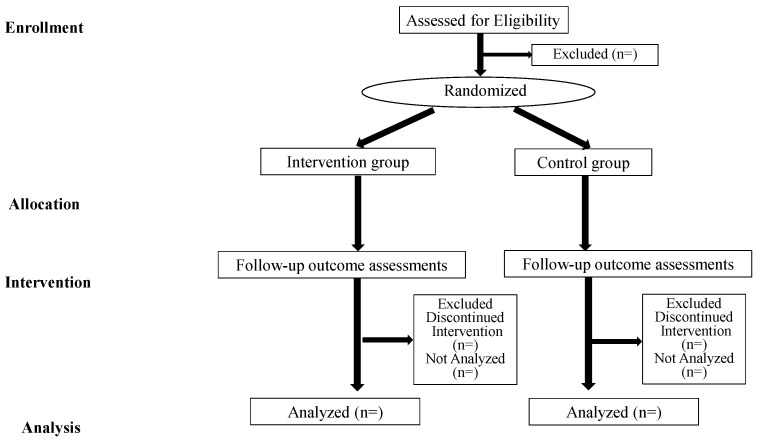
CONSORT flowchart.

To avoid contamination between the two groups, we will conduct the VR or video-watching simulation at the same time in separate rooms. Additionally, we will prevent the participants of the definitive RCT in 2023 from having any information. Therefore, all pilot study participants will be asked to keep the information about the simulation program confidential until the end of the definitive RCT in 2023. This study protocol will be based on the Standard Protocol Items: Recommendations for Interventional Trials (SPIRIT) [33] and Consolidated Standards of Reporting Trial (CONSORT) [34] in Figure 1.

### 2.2. Study Setting and Recruitment

Phase 1 and 2 studies will be conducted at the universities that the first and fourth researchers belong to in Tokyo. In Phase 2, we will distribute the research description document for recruiting participants for a pilot study in 2022 from the two universities in Tokyo, wherein the first and fourth researchers are affiliated. Nursing students will access the webform and read the documents, explaining the study’s aim and procedures and how to protect their privacy. After obtaining informed consent regarding participation in this study through the webform, they will register their e-mail address and pseudonyms and answer the baseline survey.

Additionally, the main RCT will be conducted in the spring semester class in 2023 at one university in Tokyo, to which the fourth and fifth researchers are affiliated. All students will answer the baseline and post-test evaluation as coursework. After obtaining informed consent from nursing students, they will submit their anonymous data to the researchers to prevent associating the student’s grade evaluation with the content of their data.

### 2.3. Participants

The inclusion criteria for the Phase 1 study are HVNs or PHNs that have worked in the Tokyo Metropolitan area. The exclusion criteria are HVNs or PHNs who are novice level.

The inclusion criteria for the Phase 2 study are as follows: (1) for the pilot study, nursing students will be in the third or fourth grades of the universities that the first and fourth researchers belong to in 2022; (2) for the main RCT, nursing students will be in their third grade of the university that the fourth and fifth researcher belongs to in 2023. They will have to take a home care nursing course in the spring semester of 2023. The exclusion criteria for the Phase 2 are students who will be outside of these two universities in 2023.

### 2.4. Sample Size

To consider the feasibility of the Phase 1 study, we will have interviews with less than ten HVNs or PHNs. For the Phase 2 pilot study to be feasible, we will recruit approximately 12 nursing students. In 2023, we will conduct the definitive RCT at the university, wherein the fourth and fifth researchers are affiliated. We will include approximately 80 students in their third grade at the university. The RCT will aim for a three-point intergroup difference based on a previous study [35]. The standard deviation of each group is calculated using a prior study to σ = 3.09 [35]. The number of samples required to show the superiority of the intervention group under the conditions of significance level α = 0.05 and power β = 0.8 will be expressed by the following formula (using *t*-test; Z is the standard normal distribution); N = 2[Z1 − α/2 + Z1 − β]2(σ/δ). If δ is considered X, the number of examples required is 18. Assuming a dropout rate of 20% and a limited number of students will participate in this study, the minimum number of samples required for each group will be 22.

### 2.5. Randomization

In Phase 2, eligible students will be randomly allocated to an intervention (using VR with HMD) and control group (watching the video) [1:1] by HF specializing in statistics based on an equal computer-based randomization table using a computerized random number generator.

### 2.6. The Intervention

#### 2.6.1. The Experimental Intervention

This simulation program is based on three theories that include experimental learning [36], reflective practice [37], and Jeffries’s simulation design for nursing education [38]. Based on the curriculum, we developed the goals of the class as follows: (1) understanding the lives of each subject in their homes; (2) identifying lives that will affect the subject’s health conditions; (3) considering concrete questions to identify or understand each subject’s healthcare needs (Appendix A, Table A1). We considered the life cycle, the total number of family members, and the most experienced cases in their practical training in the community and home settings. We developed three themes, scenes of simulation, and their scenarios, including (1) a household of an older person, (2) a household of an older couple, and (3) a household of parents with an infant.

Table A2 (Appendix A) shows the timeline for the simulation program and data collection. The program will be conducted in three sessions of 90 min each. The length of each VR clip is approximately four minutes. All faculty members are well-trained and will use the same procedural guidelines for conducting the simulation program. We will show a mini-test with five questions confirming students’ knowledge about each theme. The researchers will explain the goals and objectives of the class and conduct a briefing session for each case. All participants will experience each simulation.

In the first simulation session, students will watch a VR clip and assess the factors affecting the subject’s health. Other students will become observers and take notes of the first student’s good and improvement points for the second simulation session by watching the monitor in the same room. We will record eye-tracking data of the intervention group participants with their permission to compare with the professional nurses.

After watching the VR clip, we will conduct a debriefing session. First, the students who will run the simulation will share their experiences with others. All the students will then provide their positive feedback and discuss how to improve the next simulation within 15 min. The researcher will integrate their experiences and opinions to enhance clinical reasoning skills for the next simulation. After the first debriefing session, the second and third simulation and debriefing sessions will be repeated with the same procedure.

#### 2.6.2. The Control Intervention

The control group will watch three videos, including the same contents and scenarios. The procedure of briefing and debriefing will be the same as the intervention group.

### 2.7. Instruments and Measures

The participants will need approximately 10 min to answer the baseline survey and about 20 min for the post-test survey. We will use three scales developed by the National League for Nursing (NLN) to evaluate simulation education for nursing students and confirm reliability and validity for use in English [39]. We will translate this English scale to Japanese and conduct back translation as the scale development procedure. The participants will answer these scales post-intervention.

#### 2.7.1. Demographic Data

The participants’ demographic data will include age, gender, grade level, experience in home visitation through on-campus exercises or practical training, and previous experience using VR as a baseline.

#### 2.7.2. Nursing Students’ Self-Assessment of Clinical Reasoning Skills

As the primary outcome, we will measure self-assessment of clinical reasoning skills at the baseline and post-intervention stages by using original items initially developed. The participants will answer according to their understanding level and status of implementation on a scale ranging from 0 (unable to do) to 10 (adequately able to do) based on the national guideline for nursing education [40,41], core competencies, and graduation goals in bachelor’s degree of nursing education [42], and evaluation indicators of PHN education [43].

#### 2.7.3. Student Satisfaction and Self-Confidence in Learning©

The secondary outcome includes 13 items assessing the “student satisfaction” (five items) and “self-confidence” (eight items) in simulation learning will be measured on a 5-point Likert scale: “1 (strongly disagree), 2 (disagree with the idea), 3 (undecided), 4 (agree), 5 (strongly agree)” [44].

#### 2.7.4. Simulation Design Scale© (Student Version)

The third outcome will assess 20 items of the “simulation design elements, including objectives or information, support, problem-solving, feedback, and fidelity” [45]. This scale will be measured on a 5-point Likert scale and NA: “1 (strongly disagree), 2 (disagree), 3 (undecided), 4 (agree), 5 (strongly agree), NA (not applicable)”. Additionally, the importance of items will be measured on a 5-point Likert scale, ranging from 1 to 5: “1 (not important), 2 (somewhat important), 3 (neutral), 4 (important), and 5 (very important).”

#### 2.7.5. Educational Practices Questionnaire© (Student Version)

This scale, including four dimensions within 16 items, “active learning, collaboration, diverse ways of learning, and high expectations,” would be used by faculty members for measuring the best simulation [46]. Assessing the educational practices will be measured using a 5-point Likert scale: “1 (strongly disagree), 2 (disagree), 3 (undecided), 4 (agree), 5 (strongly agree), and NA (not applicable).” Additionally, the importance of each item will be measured on a 5-point Likert scale: “1 (not important), 2 (somewhat important), 3 (neutral), 4 (important), and 5 (very important).”

#### 2.7.6. Usability Metric for User Experience (UMUX)-LITE

We will ask the intervention group about the system usability at the post-intervention stage, including two items; “This system’s capabilities meet my requirements” and “This system is easy to use,” and the score range is “0 (strongly disagree) to 7 (strongly agree)” [47].

#### 2.7.7. Igroup Presence Questionnaire (IPQ)

We will ask the intervention group about the IPQ scale post-intervention. IPQ scale will measure the realness of being in a virtual environment using 14 items by using a “0 (strongly disagree) to 7 (strongly agree)” [48].

#### 2.7.8. VR Sickness

We will evaluate VR sickness levels with a 4-point Likert scale, “1 (not feel), 2 (little feel), 3 (sense), and 4 (strongly feel),” among the intervention group. Additionally, they will identify the scene of each clip when they feel VR sickness.

#### 2.7.9. Free Description

In the Phase 1 study, HVNs and PHNs will comment on the assessment points of each subject and clip. Additionally, they will describe the program’s appropriateness for nursing education and improvement points. In the Phase 2 study, all students will write down their assessment and reason for it and will be free to describe their thoughts.

### 2.8. Data Management

This study will not involve any potentially harmful interventions, and a data monitoring committee will not be established. Researchers CH and YS will develop a data-collecting form and manage all the data. In case of trouble, they will share that information with the research team. Researchers CH and YS will provide all data to the primary researcher KYM and HF, an expert in medical statistics, via a password-locked Microsoft Excel file. All researchers will monitor the data collection procedure for quality assurance.

### 2.9. Statistical Analysis

The data will be analyzed using IBM SPSS for Windows (version 25; IBM Corp, Armonk, NY, USA), and a value of *p* < 0.05 will be statistically significant. Descriptive statistics will analyze the intergroup differences between baseline and post-test data using the Mann-Whitney U-test and the chi-square/Fisher’s exact test. Furthermore, we will calculate Cronbach’s alpha coefficient to examine the internal reliability of the three scales developed by the NLN, UMUX-LITE, and IPQ. In the definitive RCT, we will analyze the data based on the intention-to-treat principle. The number of times and duration of locations seen by participants will be calculated as eye-tracking data.

We will analyze the free description of all participants. We will code each data using the Microsoft Excel worksheet and explore their similarities. We will improve the simulation program based on their negative comments.

### 2.10. Ethical Considerations

The primary researcher’s Institutional Review Board (IRB) approved the study protocol on 31 July 2022 (ID: 2022111NI). Additionally, the other four IRBs approved the study protocol as well. This study is designed based on the Declaration of Helsinki. We have registered the protocol of the main RCT in the University Hospital Medical Information Network Clinical Trials Registry (UMIN-CTR) following the International Committee of Medical Journal Editors (No. UMIN000049037, 27 September 2022). We will explain this study’s aim and procedures and protect each participant’s privacy. Only the HVNs, PHN, and nursing students with informed consent will participate in this study. We will ensure the privacy of all participants by using pseudonyms throughout the study. This study will not collect personal information as data. Especially for the students, we will explain that we will not associate the student’s grade evaluation with the content of their research responses. There will be no disadvantage to the subject if they do not participate in the study.

## 3. Conclusions

The VR nursing simulation program will help improve nursing students’ clinical reasoning skills and confidence to assess persons’ healthcare needs in home settings. Additionally, the VR simulation will show a new concept that focuses on evaluating the subjects’ healthcare needs based on their lives that will affect them. The Phase 1 validation study and Phase 2 pilot RCT will provide vital feedback to improve the feasibilities and procedures of the definitive RCT in 2023.

## Data Availability

Not applicable.

## Appendix A

**Table A1:** Outline of the themes of simulation programs.

Subjects	Goals of the Class	The Layout of Each Subject’s Home	Scenarios of Simulation
A household of an older person	(1) Understanding the lives of each subject in their homes. (2) Identifying living conditions that will affect the subject’s health conditions. (3) Considering concrete questions to identify or understand each subject’s health and life.		An 82-year-old female. Past health history: acute myocardial infarction, chronic heart failure, hypertension, obesity, and a history of falls at her home. Hospitalization: no history of heart failure within five years due. Her daughter informed us that she had recently forgotten to take her medication. She recently fell and sustained an abrasion on her forehead while walking from the kitchen to the Japanese-style room.
A household of the older couple			An 80-year-old male. His past health history: (1) hypertension (since age 55), (2) Parkinson’s disease (since age 69): the third grade of Hoehn and Yahr. Medication: (1) L-dopa due to having on-off phenomenon, muscle stiffness, and slowness of movement; (2) antihypertensive medication. His wife is 79 years old. Her past health history: (1) type 2 diabetes, (2) a history of falling at home, fracturing her right femoral neck, and undergoing artificial head replacement surgery. After the surgery and rehabilitation, she was discharged from the hospital with a cane.
A household of parents with an infant			A 41-year-old male and 39-year-old female, and an infant. Father’s work: a firefighter with repeating day and night shifts. Mother’s status: taking on the first maternity leave. Her past health condition: none. Her health condition: lack of sleep. She is almost solely responsible for caring for her infant and doing her best to provide good care for her baby.

**Table A2:** Timeline for the simulation program.

Program	Contents	Timetable	Measurements
Before starting the class	The table randomly assigning students to the intervention of control groups is posted near the classroom door.	—	—
	Before entering the room, the students will receive “VR” or “Video” stickers from the assigned group. The students will write their pseudonyms on each sticker and put it on their left chest.	15 min	—
	The intervention group will practice how to operate the VR with HMD.	30 min	—
	Baseline assessment	10 min	Demographic data Self-assessment of clinical reasoning skills (pre-test)
Briefing: theme 1 (household of an older person)	The researchers will explain the goals, themes, and overview of the cases and their tasks to the students.	3 min	—
Mini test 1 (theme 1)	Assessing the students’ knowledge regarding each case.	5 min	Mini test (5 questions)
Simulation 1 (theme 1)	The first group of students will watch a VR clip with HMD or video. Other students will observe their simulation and write down what they will observe and their reasons on the simulation exercise sheet.	4 min × 3	(Recording eye-tracking data of the intervention group participants with their permission.) Fill in the simulation exercise sheet while watching the clip.
Debriefing 1 (theme 1)	The students who mainly have experienced the simulation program will share their experience with observers. Observers will give feedback and their comments for improving their next simulation session. The researcher will integrate their experiences and opinions to enhance clinical reasoning skills for the next simulation.	First debriefing: within 20 min Second and third debriefing: 15 min	Fill in the simulation exercise sheet while debriefing.
(Break)		(10 min)	—
Briefing 2 and 3 (theme 2: households of the older couple; theme 3: households of parents with an infant)	The researchers will explain the goals, themes, and overview of the cases and their tasks to the students.	3 min	—
Mini-test 2 and 3	Assessing students’ knowledge regarding each case.	5 min	Mini-test includes 5–6 questions.
Simulation 2 and 3 (theme 2 and 3)	Simulations × 3 (the same method was used for simulation 1.)	4 min × 3	(Recording eye-tracking data of the intervention group participants with their permission.) Fill in the simulation exercise sheet while watching the clip.
Debriefing 2 and 3 (theme 2 and 3)	Debriefing × 3 (the same method was used for debriefing 1.)	First debriefing: within 20 min Second and third debriefing: 15 min	Fill in the simulation exercise sheet while debriefing.
Summary	The researcher will summarize the simulation.	20 min	—
After the class	Follow-up assessment Only students, with informed consent, will answer the post-test survey, mini-test, and simulation exercise sheet.	—	Self-assessment of clinical reasoning skills (post-test) Student Satisfaction and Self-Confidence in Learning© Simulation Design Scale© (Student Version) Educational Practices Questionnaire© (Student Version) Questionnaire on Educational Practices UMUX-LITE (for only the intervention group) IPQ (for only the intervention group) Free description

Note: © = copyright mark; VR = virtual reality; HMD = head-mounted display; UMUX-LITE = the usability metric for user experience; IPQ = the igroup presence questionnaire.
